# In Vitro and In Silico Analysis of Differential Antibacterial Activity of Pomegranate Polyphenols Against Gram-Positive and Gram-Negative Bacteria

**DOI:** 10.3390/antibiotics14090912

**Published:** 2025-09-10

**Authors:** Relja Suručić, Maja Travar, Tatjana Kundaković Vasović, Jelena Radović Selgrad, Ljiljana Suručić, Milan Momčilović, Miloš P. Stojiljković, Ranko Škrbić

**Affiliations:** 1 Department of Pharmacognosy, Faculty of Medicine, University of Banja Luka, 78000 Banja Luka, The Republic of Srpska, Bosnia and Herzegovina; 2 Department of Microbiology, Faculty of Medicine, University of Banja Luka, 78000 Banja Luka, The Republic of Srpska, Bosnia and Herzegovina; 3 Department of Pharmacognosy, Faculty of Pharmacy, University of Belgrade, 11221 Belgrade, Serbia; 4 Department of Organic Chemistry, Faculty of Medicine, University of Banja Luka, 78000 Banja Luka, The Republic of Srpska, Bosnia and Herzegovina; 5 Department of Chemistry, Faculty of Sciences and Mathematics, University of Niš, Višegradska 33, 18000 Niš, Serbia; 6 Department of Pharmacology, Toxicology and Clinical Pharmacology, Faculty of Medicine, University of Banja Luka, 78000 Banja Luka, The Republic of Srpska, Bosnia and Herzegovina; 7 Centre for Biomedical Research, Faculty of Medicine, University of Banja Luka, 78000 Banja Luka, The Republic of Srpska, Bosnia and Herzegovina; 8 Academy of Science and Arts of the Republic of Srpska, 78000 Banja Luka, The Republic of Srpska, Bosnia and Herzegovina; 9 Department of Pathologic Physiology, I.M. Sechenov First Moscow State Medical University, 119435 Moscow, Russia

**Keywords:** pomegranate peel extract, polyphenols, antimicrobial activity, molecular docking, Gram-positive and Gram-negative bacteria, porin channel transport

## Abstract

Background/Objectives: This study investigates the antimicrobial properties of pomegranate peel extract (PoPEx) and its major polyphenolic constituents against Gram-positive and Gram-negative bacteria, employing six clinical isolates of *Staphylococcus aureus* and five isolates of *Escherichia coli*. The study further aims to elucidate mechanisms of action through molecular docking and transport studies. Methods: Chemical composition was analyzed using liquid chromatography–mass spectrometry (LC–MS). Antimicrobial activity was determined by the broth microdilution method. Molecular docking was performed with the AutoDock Vina algorithm, and transport studies through porin channels were carried out using Caver software. Results: PoPEx showed stronger activity against Gram-positive (MICs 15.62–500.00 μg/mL) than Gram-negative bacteria (MICs 125.00–500.00 μg/mL). Punicalagin was most active against *S. aureus*, while gallic acid was most effective against *E. coli*. Docking revealed high affinities of punicalagin and punicalin, whereas transport studies highlighted the advantage of smaller phenolics like gallic acid in crossing porins. Conclusions: Larger tannins exhibited strong target binding but limited porin permeability, reducing efficacy in Gram-negative bacteria. These findings provide insights into structure–activity relationships of pomegranate polyphenols and support their potential as natural antimicrobial agents.

## 1. Introduction

Antimicrobial resistance represents one of the most significant global health challenges of the 21st century. The rapid emergence and spread of multidrug-resistant bacterial pathogens threaten to undermine decades of progress in infectious disease management, with the World Health Organization identifying antimicrobial resistance as one of the top ten global public health threats facing humanity [1]. This crisis has prompted an urgent need for novel antimicrobial agents with unique mechanisms of action that can overcome existing resistance mechanisms.

In this context, natural products derived from plants have garnered renewed interest as potential sources of antimicrobial compounds. Medicinal plants have been utilized throughout human history for their therapeutic properties, including antimicrobial effects. The rich diversity of secondary metabolites produced by plants represents an extensive reservoir of bioactive compounds with potential pharmaceutical applications.

Among these, pomegranate (*Punica granatum* L.) has a particularly distinguished history in traditional medicine systems across multiple cultures. Ancient civilizations in the Mediterranean, Middle East, and Asia have documented the use of various parts of the pomegranate plant for treating infections, diarrhea, parasitic diseases, and other ailments [2]. Modern scientific investigations have substantiated many of these traditional applications, revealing pomegranate’s broad spectrum of biological activities, including antimicrobial, antioxidant, anti-inflammatory, antiviral and anticancer properties [3,4,5].

While the edible arils of pomegranate fruits are widely consumed and studied, the peel, which constitutes approximately 26–30% of the total fruit weight, has emerged as a particularly rich source of bioactive compounds [6]. Pomegranate peel, often discarded as agricultural waste, contains significantly higher concentrations of polyphenolic compounds than the edible portions, making it an attractive subject for valorization research [7]. The polyphenol complex of pomegranate peel has more pronounced antioxidant and anti-inflammatory properties than other parts of the fruit. The most important active components of pomegranate peel, which are found predominantly in this plant, are punicalagin and punicalin followed by ellagic acid and gallic acid [5,8]. These polyphenolic compounds have demonstrated the most pronounced therapeutic effects in various studies. The antimicrobial activity of pomegranate peel extract (PoPEx) has been attributed to its rich polyphenolic content, particularly ellagitannins like punicalagin and punicalin, as well as phenolic acids such as ellagic acid and gallic acid [9]. These compounds exhibit complex mechanisms of action against bacterial pathogens, including membrane disruption, enzyme inhibition, and interference with bacterial cell division processes [10]. Understanding the precise molecular interactions between these polyphenolic compounds and bacterial targets is essential for elucidating their antimicrobial mechanisms and potential applications. However, the development of plant-derived antimicrobials faces significant challenges that extend beyond simple identification of bioactive compounds. Critical gaps remain in our understanding of how molecular size, solubility, and aggregation behavior of natural compounds influence their antimicrobial efficacy in complex biological systems. The central hypothesis of this study is that the differential antimicrobial activity of pomegranate polyphenols against Gram-positive and Gram-negative bacteria is primarily governed by molecular size-dependent membrane permeability constraints, rather than inherent differences in target binding affinity. We propose that while large ellagitannins like punicalagin demonstrate superior binding affinity to bacterial targets through multiple hydrogen bonding and hydrophobic interactions, their limited diffusion through Gram-negative outer membrane porins significantly reduces their practical antimicrobial effectiveness. Conversely, smaller phenolic compounds such as gallic acid may exhibit lower target binding affinity but achieve superior antimicrobial activity against Gram-negative bacteria due to enhanced membrane permeability [11]. This hypothesis guided our study design and raised several key research questions, namely how the molecular weight and structural complexity of pomegranate polyphenols correlate with their antimicrobial potency across different bacterial envelope architectures, what the relative contribution of target binding affinity versus membrane permeability is to overall antimicrobial effectiveness, and whether computational modeling of porin channel transport can reliably predict the practical antimicrobial activity of polyphenolic compounds.

Molecular docking studies have provided valuable insights into the interactions between plant-derived compounds and bacterial targets. In particular, ellagitannins have shown potential to interact with pharmacologically relevant targets, including bacterial enzymes and membrane proteins, supporting their possible role as antimicrobial agents [4,5,12,13]. In the context of antimicrobial research, several key bacterial proteins have been identified as potential targets for inhibition. Penicillin-binding proteins (PBPs) play a crucial role in bacterial cell wall synthesis, specifically in the cross-linking of peptidoglycan stem peptides. These proteins have been validated as effective antimicrobial targets through the clinical success of β-lactam antibiotics, which mimic the D-alanine-D-alanine dipeptide substrate of PBPs and form a stable acyl-enzyme complex with the active site serine, thereby inhibiting transpeptidation and leading to cell wall weakening and eventual lysis [14]. In *S. aureus*, acquisition of the *mecA* gene enables production of penicillin-binding protein 2a (PBP2a)—a β-lactam-insensitive transpeptidase that continues cell wall crosslinking even in the presence of antibiotics. This low-affinity PBP’s active site remains “closed” to β-lactams, underpinning the methicillin-resistant *S. aureus* (MRSA) phenotype and making PBP2a a prime target for in silico docking studies and novel inhibitor design. Likewise, *E. coli* relies on penicillin-binding protein 3 (PBP3) for cell division, and many β-lactams (penicillins, cephalosporins, etc.) act by binding this essential enzyme. Mutations in *E. coli* PBP3—for instance, insertion of extra amino acids near PBP3’s active site—can dramatically raise the minimum inhibitory concentrations of PBP3-targeted drugs, confer resistance while still retain enzymatic function. These resistance mechanisms have motivated numerous computational modeling and drug design efforts aimed at PBP2a and PBP3, seeking new compounds (including non-β-lactam inhibitors) that can effectively bind these altered PBPs and overcome bacterial resistance [15,16].

Another important target is MurA (UDP-N-acetylglucosamine enolpyruvyl transferase), which catalyzes the first committed step in bacterial cell wall peptidoglycan synthesis. MurA transfers enolpyruvate from phosphoenolpyruvate (PEP) to UDP-N-acetylglucosamine, initiating the pathway for peptidoglycan formation. This enzyme is the target of the clinically used antibiotic fosfomycin, which binds competitively to the active site and undergoes a ring-opening nucleophilic reaction [17]. The essential nature of MurA and its high conservation across bacterial species make it an attractive target for antimicrobial development.

FtsZ (Filamenting temperature sensitive mutant Z) represents a third significant target for antimicrobial intervention. This bacterial homolog of eukaryotic tubulin is essential for bacterial cell division, forming a ring-like structure (Z-ring) at the prospective division site that serves as a scaffold for the divisome complex. Inhibition of FtsZ results in the complete blockage of the division system, leading to bacteriostatic or bactericidal effects [18]. FtsZ contains several potential binding sites for inhibitors, including the GTP-binding site in the globular core and an interdomain site located in a cleft between the C-terminal domain and the H7 helix.

The differential susceptibility of Gram-positive and Gram-negative bacteria to antimicrobial agents, including plant-derived compounds, is largely attributed to structural differences in their cell envelopes. Gram-positive bacteria possess a thick peptidoglycan layer surrounding the cytoplasmic membrane, while Gram-negative bacteria have a thinner peptidoglycan layer sandwiched between the cytoplasmic membrane and an outer membrane composed of lipopolysaccharides. This outer membrane serves as a permeability barrier, restricting the entry of many antimicrobial compounds [19]. Additionally, the presence of porin channels in the outer membrane of Gram-negative bacteria controls the passive diffusion of hydrophilic molecules, with size limitations that prevent the passage of larger compounds, while allowing smaller molecules to enter [20].This size-exclusion effect may explain why computational docking studies often fail to predict actual antimicrobial activity—while large ellagitannins may show excellent binding affinity to intracellular targets, their inability to reach these targets in sufficient concentrations renders them ineffective in practice.

The present study aims to comprehensively evaluate the antimicrobial effects of PoPEx, with a particular emphasis on the role of its main polyphenolic constituents and their differential activity against 6 clinical isolates of *S. aureus* (representing Gram-positive bacteria) and 5 clinical isolates of *E. coli* (representing Gram-negative bacteria). Testing novel antimicrobials against a diverse set of clinical isolates is essential for accurately assessing their therapeutic potential and predicting their effectiveness in real-world scenarios. Unlike standard laboratory strains, clinical isolates often harbor acquired resistance mechanisms and exhibit significant genetic and phenotypic diversity, reflecting the complexity of infections encountered in healthcare settings. Evaluating antimicrobial activity across multiple strains enables the identification of resistance patterns, supports the development of broad-spectrum agents, and informs rational drug design. This approach is crucial for addressing the growing global challenge of antimicrobial resistance [21]. What distinguishes this study from previous investigations is our integrated approach that combines in vitro assays on clinical isolates with computational modeling of membrane permeability. Unlike earlier studies that focused primarily on MIC determination or molecular docking in isolation, we provide a mechanistic framework that explains apparent contradictions between computational predictions and experimental observations. Our work specifically addresses the critical gap in understanding how physicochemical properties of polyphenolic compounds influence their practical antimicrobial effectiveness, moving beyond simple structure–activity relationships to consider bioavailability and delivery constraints.

We also acknowledge limitations, as we did not evaluate different solvents or extraction conditions, which may significantly influence both yield and biological activity of pomegranate polyphenols. Previous research has shown that extraction efficiency can vary greatly depending on solvent selection, temperature, time, and solid-to-liquid ratios, leading to wide variability in reported MIC values for PoPEx. Furthermore, high-molecular-weight compounds like punicalagin are prone to aggregation and precipitation in aqueous solutions at concentrations typically used in antimicrobial testing, which can reduce bioavailability, alter interaction kinetics, and underestimate true antimicrobial activity. These issues should be addressed in future studies, ideally complemented by comprehensive toxicological evaluations.

Our ultimate goal is to establish a predictive framework that can guide the rational design of polyphenol-based antimicrobials with optimized molecular properties for enhanced membrane permeability while maintaining target binding affinity.

## 2. Results

### 2.1. Chemical Composition of Pomegranate Peel Extract

The PoPEx obtained through methanol extraction yielded a fine reddish-brown powder with a characteristic astringent odor. The extraction process resulted in a yield of 28.7 ± 1.2% (*w*/*w*) based on the dry weight of pomegranate peel powder.

**Figure 1:**
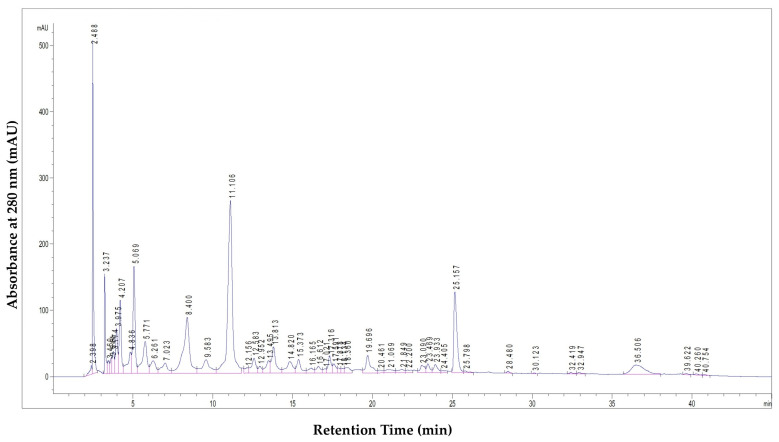
LC–MS chromatogram of the tested PoPEx sample, illustrating the major identified polyphenolic compounds.

Chemical analysis of PoPEx revealed the presence of several polyphenolic compounds, with hydrolyzable tannins and phenolic acids being the predominant classes (Figure 1, Table 1).

The chromatographic profile displayed distinct peaks corresponding to the major bioactive compounds. These peaks were identified by comparing their retention times and UV spectra with those of commercial standards, or tentatively assigned based on literature data for compounds without available standards. The quantitative analysis of these compounds is presented in Table 1.

Punicalagin, present as α and β isomers, was identified as the most abundant polyphenolic compound in PoPEx, with a total concentration of 623.91 mg/g of dry extract. Other prominent compounds included punicalin isomers (10.44 mg/g and 12.14 mg/g), pedunculagin I (12.5 mg/g), galloyl-HHDP-hexoside (5.71 mg/g), tellimagrandin I (2.74 mg/g), ellagic acid hexoside (6.01 mg/g), and free ellagic acid (26.54 mg/g).

The structural characteristics of these polyphenolic compounds—particularly the abundance of hydroxyl groups and their capacity to form complexes with proteins and other macromolecules—significantly contribute to their potent biological activity

### 2.2. In Vitro Antimicrobial Activity Results

The antimicrobial activity of PoPEx and its major polyphenolic compounds was evaluated against 6 clinical isolates of *S. aureus* as representative Gram-positive bacteria, and 5 clinical isolates of *E. coli* as representative Gram-negative bacteria. Testing these clinical isolates, rather than laboratory reference strains alone, is critical for assessing the real-life effectiveness of natural antimicrobials. Clinical isolates often possess unique resistance mechanisms absent in standard strains, making them more relevant for evaluating antimicrobial efficacy. Antibiotic susceptibility testing of the clinical isolates of *S. aureus* and *E. coli* is presented in Table 2.

All *S. aureus* isolates exhibited sensitivity to cefaclor, ceftriaxone and vancomycin, whereas resistance was observed in some strains to penicillin, ampicillin, erythromycin, gentamicin and ciprofloxacin. In contrast, the *E. coli* isolates demonstrated uniform resistance to piperacillin and partial resistance to ampicillin, amoxicillin/clavulanic acid and trimethoprim/sulfamethoxazole, while remaining susceptible to most cephalosporins, aminoglycosides, and carbapenems. These results indicate that both *S. aureus* and *E. coli* isolates show clinically relevant resistance profiles, thereby justifying their selection as representative drug-resistant strains for the present study. The minimum inhibitory concentrations (MICs) determined for each tested sample against *S. aureus* are presented in Table 3.

PoPEx and its major polyphenolic constituents showed significant inhibitory effects against the six clinical *S. aureus* isolates tested. PoPEx exhibited consistent, moderate activity with MIC values between approximately 20.8 and 31.3 µg/mL across these isolates. Among the individual compounds, punicalagin was the most potent, with uniformly low MICs (15.6–31.3 µg/mL). In fact, punicalagin achieved the lowest MIC observed against *S. aureus* (15.62 µg/mL in several isolates), highlighting it as the strongest antimicrobial agent in the extract’s profile. Punicalin also inhibited *S. aureus* but required higher concentrations than punicalagin (MICs mostly around 62.5 µg/mL, with a slight isolate-to-isolate variation, lowest ~52 µg/mL). Gallic acid and ellagic acid, the smaller phenolic constituents, showed substantially weaker activity in comparison: gallic acid’s MIC was 125 µg/mL for most *S. aureus* isolates (one isolate showed a slightly higher MIC, ~167 µg/mL), while ellagic acid consistently required 250 µg/mL to inhibit *S. aureus*. These higher MIC values for the phenolic acids indicate lower potency, though they still demonstrate measurable antibacterial effect at sufficient concentrations. However, pedunculagin was the least effective compound against *S. aureus*, with MIC values at or above the highest concentration tested (500 µg/mL) across all isolates, indicating minimal inhibitory activity. The results indicate that the antimicrobial activity of PoPEx against Gram-positive bacteria is mainly attributable to its major ellagitannins, punicalagin and punicalin, while smaller phenolic compounds such as gallic acid, ellagic acid, and pedunculagin contribute only marginally. In contrast to the results with *S. aureus*, the MIC values obtained for the six *E. coli* clinical isolates (Table 4) were markedly higher, reflecting a lower susceptibility of Gram-negative bacteria to these natural compounds.

PoPEx still displayed inhibitory activity against *E. coli*, but at concentrations around an order of magnitude higher than for *S. aureus* (MICs generally in the 200–250 µg/mL range, with most values at 250 µg/mL and the lowest around 208 µg/mL). This moderate activity of the whole extract suggests that *E. coli*’s cellular defenses (such as the outer membrane barrier) limit the effectiveness of PoPEx. The individual polyphenols followed a similar trend of reduced potency against *E. coli.* The ellagitannins punicalin and punicalagin, which were quite effective on *S. aureus*, required much higher concentrations to inhibit *E. coli*. Punicalagin’s MICs ranged roughly from 250 to 333 µg/mL across the isolates (with some variation between strains), while punicalin was even less effective, with MICs typically at 500 µg/mL. Pedunculagin again displayed the poorest activity: *E. coli* growth was essentially unaffected at 500 µg/mL, meaning its MIC for all isolates exceeded the 500 µg/mL testing limit. Overall, the larger hydrolyzable tannins (punicalagin, punicalin, pedunculagin) displayed limited activity against Gram-negative strains, whereas the smaller phenolic acids (gallic and ellagic) showed comparatively better effects on *E. coli*, though their MIC values still remained high in absolute terms. Gallic acid in particular inhibited most *E. coli* isolates at 125 µg/mL, which is lower than the MICs of any ellagitannin in this species. This may suggest that a small molecule like gallic acid can penetrate or evade defenses more effectively than the bulkier tannins. Ellagic acid exhibited MIC values of around 250 µg/mL against *E. coli* for most isolates, while two strains were more susceptible.

### 2.3. Molecular Docking Study Results

In order to evaluate the potential mechanisms underlying the observed antimicrobial activity, clinically relevant bacterial targets were selected for molecular docking studies. Specifically, penicillin-binding proteins (PBP), UDP-N-acetylglucosamine enolpyruvyl transferase (MurA), and filamenting temperature-sensitive mutant Z (FtsZ) were chosen due to their crucial roles in bacterial cell wall biosynthesis and division processes. Crystal structures of these targets from both Gram-positive (*S. aureus*) and Gram-negative (*E. coli*) microorganisms were used to ensure a comprehensive analysis.

These targets are particularly relevant to the observed in vitro antimicrobial assays and may help explain the higher susceptibility of Gram-positive *S. aureus* compared to Gram-negative *E. coli*. This difference in susceptibility can be attributed to structural variations in the bacterial cell envelope. Gram-positive bacteria possess a thick peptidoglycan layer without an outer membrane, which provides direct access for many antimicrobial agents. In contrast, Gram-negative bacteria feature a thin peptidoglycan layer surrounded by an outer membrane that serves as a permeability barrier, limiting the entry of many hydrophilic and bulky molecules, including polyphenolic compounds.

Results of molecular docking analysis are presented in Table 5.

**Figure 2:**
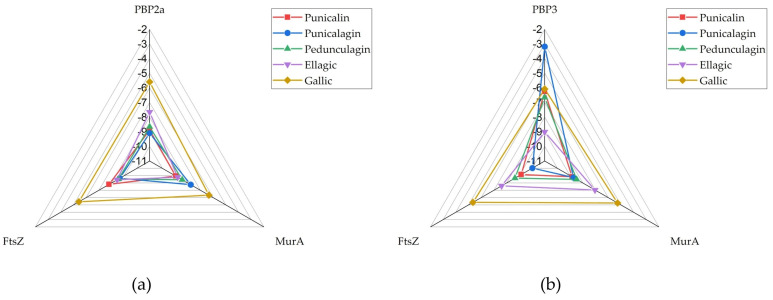
RADAR chart presenting docking scores of tested compounds against targets: (**a**) PBP2a, (**b**) PBP3.

Except for FtsZ, where punicalagin consistently exhibited the highest binding affinity for the structures derived from both Gram-positive (−8.648 kcal/mol) and Gram-negative (−10.031 kcal/mol) microorganisms, the other polyphenolic compounds demonstrated variable affinities across the different bacterial targets. The results were also presented using radar charts (Figure 2) to provide a clearer and more visual comparison of binding affinities and to better illustrate emerging trends. Compact, centered polygons indicate strong, broad-spectrum binding, while larger, outward-reaching shapes show weaker binding and target-specific differences. The size and shape of each polygon clearly show which compounds have the strongest or weakest binding. This easy-to-understand chart turns numeric binding energy data into simple “fingerprints,” making it quick and easy to see which compounds have the highest binding affinity and how they interact with different targets.

In the *S. aureus* model (Figure 2a), the radar chart illustrates that punicalin (red), punicalagin (blue) and pedunculagin (green) consistently exhibit favorable docking scores across all three targets, maintaining values well within the threshold dictated by the binding affinity of confirmed inhibitors under the same experimental conditions, indicative of strong predicted binding to PBP, MurA, and FtsZ. Ellagic acid (violet) shows a more varied profile: while it binds MurA and FtsZ with high affinity, its binding to PBP2a (*S. aureus*) is weaker. The pronounced outward extension of the violet shape along the PBP2a axis corresponds to a docking score of −7.65 kcal/mol, making it a clear outlier of lower affinity within an otherwise strong multi-target profile. Gallic acid (orange), as the smallest molecule, consistently shows the lower binding affinity of the four compounds, with the violet plot extending further outward on all three axes.

In the *E. coli* model (Figure 2b), the radar plots for the smaller molecules, gallic acid and ellagic acid, show broader shapes for FtsZ and MurA, reflecting weaker predicted interactions. In contrast, punicalin and punicalagin are more centrally positioned among the tested compounds for these targets, followed by pedunculagin. However, their affinity for PBP3 is generally lower, particularly in the case of punicalagin. The structural differences among the compounds are more pronounced in this model, with gallic and ellagic acid exhibiting significantly lower binding affinities across all targets. While punicalin displays slightly narrower radar arms compared to the *S. aureus* case—suggesting somewhat stronger binding in the Gram-negative environment—the overall binding affinities of the smaller polyphenols are reduced.

**Figure 3:**
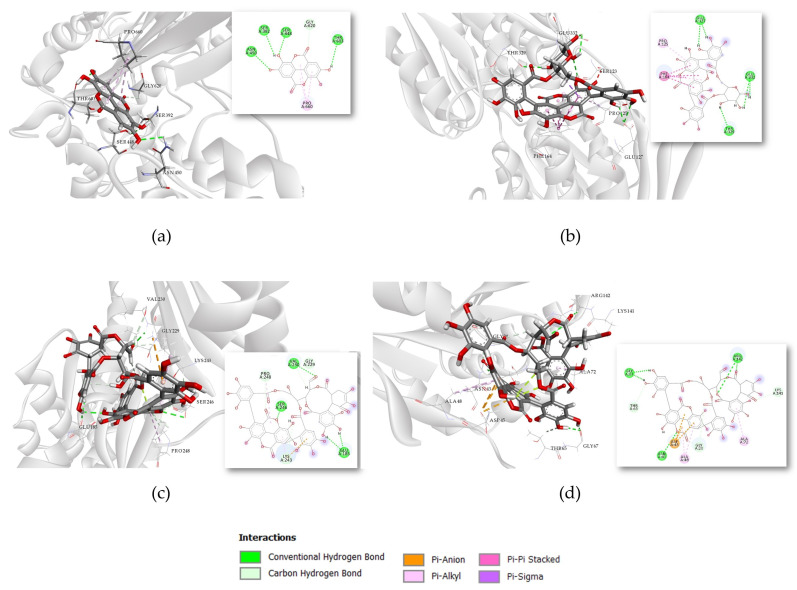
Binding interactions of compounds with the highest affinity for the tested targets in 3D and 2D presentation: (**a**) Ellagic acid binding to PBP3 (PDB ID: 3PBR), (**b**) Punicalin binding to MurA (UniProt ID: P84058), (**c**) Punicalagin binding to FtsZ (PDB ID: 3VOB), (**d**) Punicalagin binding to FtsZ (PDB ID: 6UMK).

Individual interactions of compounds with the highest affinity for the studied targets were further investigated. Punicalagin exhibited the highest affinity for PBP2a, with a binding energy of −9.09 kcal/mol. As shown in Figure 3a, the complex with PBP2a is stabilized through conventional hydrogen bonds with residues Ser 149, Thr 216, Asp 295 and Tyr 373.

The most stable complex with MurA, with a binding energy of −8.951 kcal/mol, was observed for punicalin. Its interactions with residues Glu 127, Thr 329, and Glu 332 also involve conventional hydrogen bonding. Punicalagin is the only compound to show the highest affinity for the same target (FtsZ) in both Gram-positive and Gram-negative structures, as shown in Figure 3c,d, respectively. The most stable complex was recorded for FtsZ from the Gram-negative strain, it is stabilized through hydrogen bond interactions with Asn 43, Gly 67, and Arg 142.

### 2.4. Results of Transport Studies Through Porin Channels

In order to act on its target, a ligand must reach the active site. While in molecular docking simulations we can artificially position the ligand near the active site, under physiological conditions this process is far more complex, as ligands often require transport through various mechanisms. For better understanding of differential susceptibility of Gram-positive and Gram-negative bacteria to polyphenolic compounds, computational simulations were performed to investigate their transport through the OmpF porin channel of *E. coli*. Caver Web, a computational tool used in this study, simulates the ligand transport process through protein channels. It identifies possible tunnels within the protein structure and animates the ligand’s movement through these tunnels, highlighting whether they are accessible and where potential bottlenecks occur. Consequently, Caver Web calculates an energy profile along the tunnel, pinpointing energy barriers and favorable binding points. This tool also provides a trajectory of ligand movement, with snapshots and energy plots that elucidate how the ligand navigates the tunnel.

After selecting the generated tunnel with its calculated characteristics—a bottleneck radius of 3.6 Å, length of 34.4 Å, curvature of 1.2, and throughput of 0.86—we performed simulations using Caver Web with all tested ligands. These simulations involved a series of molecular dockings along disks that make up the generated tunnel, effectively tracing the ligand’s movement from the protein surface toward the active site.

**Figure 4:**
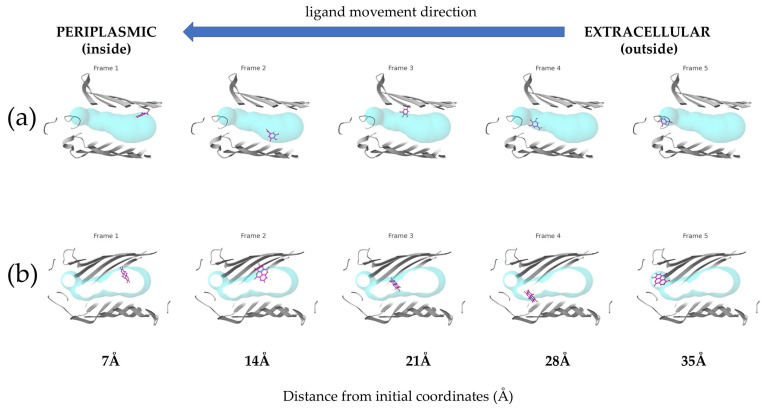
Comparative translocation of (**a**) Gallic and (**b**) Ellagic acids through the *E. coli* porin channel. The ligands are shown in violet, while the porin tunnels are represented by turquoise spheres. The blue arrow indicates the ligand movement direction from the extracellular (outside) to the periplasmic (inside) side.

However, the analysis was successfully completed only for ellagic acid and gallic acid. For the hydrolyzable tannins, no results were obtained, indicating that the ligand transport through the porin channel was not successful. The results of the transport simulations are visually presented in Figure 4, with frames created from snapshots taken at even intervals throughout the simulation.

**Figure 5:**
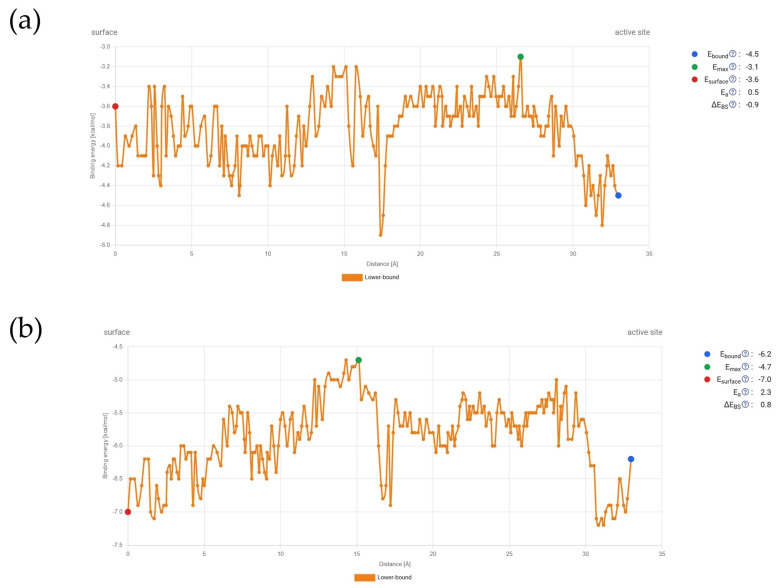
Energetic profile during transport simulation through the *E. coli* porin channel: (**a**) Gallic acid, (**b**) Ellagic acid.

Accompanying energy profiles throughout the simulation are presented in Figure 5.

These energy changes during the transport of compounds through the channel show that gallic acid’s binding energy at the start of the porin channel is higher than at the active site. Although there are energetic barriers along the way, particularly around 15 Å from the starting point, these barriers can be overcome. After that, there is a steep decrease in binding energy, followed by another rise, with E_max_ occurring around 27 Å. On the other hand, ellagic acid has a lower binding affinity at the starting point compared to the active site. Overall, the energy values are lower than those for gallic acid. Ellagic acid also encounters an energetic bottleneck at the same distance from the starting point, which can be overcome, but E_max_ is reached during the first energetic barrier before the steep decrease. Despite these differences, the profiles are quite similar.

## 3. Discussion

### 3.1. Chemical Analysis

LC–MS analysis of the methanolic pomegranate peel extract (PoPEx) confirmed that hydrolyzable tannins overwhelmingly dominate its phenolic profile. In agreement with previous phytochemical studies [22] we found punicalagin (α and β anomers combined) to be the single most abundant compound (≈623.9 mg/g of dry extract), followed by much lower amounts of punicalin (≈22.6 mg/g) and ellagic acid (≈26.5 mg/g). Other ellagitannin derivatives (e.g., HHDP-glycosides, tellimagrandins) and simple phenolic acids (e.g., gallic acid) were also detected at lower levels. For example, Čolić et al. similarly identified punicalagin, punicalin and ellagic acid as the three main peaks in PoPEx [23] with reported concentrations of 67.3, 31.9 and 25.1 mg/g, respectively. These parallels confirm that PoPEx is particularly enriched in ellagitannins. More broadly, untargeted profiling studies repeatedly single out punicalagin and its derivatives as key constituents of pomegranate peel. For instance, Man et al. used UHPLC–QTOF-MS and QQQ-MS to show that punicalagin (28.0–104.1 mg/g across cultivars) far exceeded any other phenolic in peel and similarly reported punicalin and ellagic acid as minor constituents [24]. Likewise, optimized extraction protocols (e.g., ultrasonic-assisted with 53% ethanol) have yielded PoPEx with very high punicalagin content (≈505.9 mg/g) as the peak compound [25]. In sum, our quantitative LC–MS data accord with the literature: punicalagin is consistently the major phenol in pomegranate peel extract, with punicalin and ellagic acid present at much lower levels [24].

Studies evaluating the biological effects of PoPEx have reported the presence of significant amounts of gallic acid [5,7]. The hydrolyzable tannins present in PoPEx, such as punicalagin and its monomeric analog punicalin, contain galloyl or hexahydroxydiphenoyl (HHDP) ester groups that can hydrolyze under enzymatic or acidic conditions to yield smaller phenolic molecules. In particular, galloyl-bearing tannins can liberate gallic acid upon hydrolysis of their ester bonds. Gallic acid also acts as a common precursor for the biosynthesis of punicalagin and is normally found in peel tissue [26]. Furthermore, bacteria are capable of degrading tannins [27], contributing to the release of gallic acid and other related phenolics.

### 3.2. Differential Antimicrobial Activity

The results of this study demonstrate a clear differential antimicrobial activity of PoPEx and its major polyphenolic constituents against Gram-positive and Gram-negative bacteria. PoPEx exhibited significantly higher antimicrobial potency against Gram-positive bacteria compared to Gram-negative bacteria with MIC values lower for the former. This differential susceptibility pattern aligns with previous studies on plant-derived antimicrobial agents and is likely due to fundamental differences in cell envelope architecture between Gram-positive and Gram-negative bacteria. Our findings support earlier reports demonstrating stronger activity against Gram-positive microorganisms. Additionally, prior studies have shown that, although the growth of some microorganisms was only moderately affected, their morphology underwent significant changes when exposed to subinhibitory concentrations. These alterations further suggest that the cell envelope is the primary site of action for tannins in sensitive bacterial strains [28].

The cell envelope of Gram-positive bacteria consists of a thick peptidoglycan layer (20–80 nm) surrounding the cytoplasmic membrane, with teichoic acids and proteins embedded within this structure. This relatively permeable outer layer allows for the passage of most antimicrobial compounds, including large molecules such as punicalagin (MW 1084.7 Da), to reach their targets at the cell membrane or within the cytoplasm. In contrast, Gram-negative bacteria possess a more complex cell envelope comprising a thin peptidoglycan layer (1–7 nm) sandwiched between the cytoplasmic membrane and an outer membrane composed of lipopolysaccharides, phospholipids, and proteins. This outer membrane serves as a formidable permeability barrier, restricting the entry of many antimicrobial compounds, particularly hydrophobic and high molecular weight molecules [19].

The differential activity of individual polyphenolic compounds against the two bacterial groups provides further insights into the mechanisms underlying this phenomenon. Against Gram-positive bacteria, the antimicrobial potency followed the order: punicalagin > punicalin > gallic acid > ellagic acid > pedunculagin, which correlates with the molecular complexity and number of hydroxyl groups in these compounds. Punicalagin, with its multiple hydroxyl groups and complex structure, demonstrated the strongest activity against Gram-positive bacteria, with average MIC value ≈ 19.1 µg/mL lower than those of PoPEx (≈27.8 µg/mL). This enhanced activity can be attributed to its ability to form multiple hydrogen bonds and hydrophobic interactions with bacterial targets, as evidenced by the molecular docking studies. Certain ellagitannins have demonstrated potent antibacterial activity at micromolar concentrations. Davidiin and 3-O-galloylgranatin A exhibited activity against methicillin-resistant *S. aureus* (MRSA) with MIC values of 64 μg/mL, and also showed efficacy against clinically isolated vancomycin-resistant *Enterococcus* strains, with MICs ranging from 16 to 64 μg/mL. Isorugosins, a subclass of ellagitannins, also displayed antibacterial activity against both methicillin-sensitive *S. aureus* and MRSA, with MIC values between 23 and 91 μM. Ellagic acid and punicalagin have previously shown antimicrobial effects against *S. aureus*, with reported MIC values of 12.35 and 42.11 μg/mL, respectively [9]. In our study, punicalagin exhibited comparable activity, while ellagic acid showed a significantly higher MIC. Another study reported MIC values of 12.5 μg/mL for both punicalagin and punicalin against *S. aureus*; however, our results indicated higher MIC values [29].

However, the order of antimicrobial potency was reversed for Gram-negative bacteria: gallic acid > ellagic acid > punicalagin > punicalin > pedunculagin. This inverse relationship between molecular size of compound and its antimicrobial activity against Gram-negative bacteria can be explained by the transport studies through porin channels. The OmpF porin, one of the major porins in the outer membrane of *E. coli*, forms a water-filled channel with a constriction zone of approximately 7 × 11 Å, which limits the passage of molecules based on their size, shape, and charge. Our transport simulations revealed that the smaller compounds, gallic acid (MW 170.1 Da) and ellagic acid (MW 302.2 Da), could traverse the porin channel with relatively low energy barriers, resulting in higher permeability coefficients. In contrast, the larger compounds, punicalagin, punicalin and pedunculagin encountered substantial energy barriers, significantly limiting their ability to penetrate the outer membrane through porin channels.

These findings are consistent with the “hydrophilic pathway” model for the uptake of antimicrobial compounds by Gram-negative bacteria, which suggests that hydrophilic molecules with molecular weights below approximately 600–700 Da can diffuse through porin channels, while larger or more hydrophobic molecules are largely excluded [30,31].

The synergistic effects observed in PoPEx—particularly against clinical isolates of *S. aureus*—indicate that the whole extract exhibits antimicrobial activity comparable to or even greater than that of equivalent concentrations of its individual constituents. This suggests that multiple mechanisms may contribute to its overall antimicrobial action. The diverse polyphenolic composition of PoPEx allows for simultaneous targeting of multiple bacterial structures and processes, potentially overcoming resistance mechanisms that might develop against single-target antimicrobials. Additionally, the presence of smaller polyphenolic compounds in the extract may facilitate the penetration of the outer membrane of Gram-negative bacteria, enhancing the overall antimicrobial efficacy.

### 3.3. Mechanism of Action Based on Molecular Docking Studies

Molecular docking studies offered valuable insights into the potential mechanisms of action of the polyphenolic compounds against bacterial targets. The strong binding affinities observed for the most abundant compounds suggest that key proteins in Gram-positive bacteria, particularly those involved in cell wall synthesis and cell division, may serve as primary targets underlying their antimicrobial activity

PBP2a is a penicillin-binding protein that plays a crucial role in cell wall synthesis in methicillin-resistant *S. aureus* (MRSA). Unlike other PBPs, PBP2a has a lower affinity for β-lactam antibiotics, allowing it to maintain transpeptidase activity even in the presence of these drugs, thereby conferring resistance [32].

Our docking results on the PBP2a protein target revealed that punicalagin and punicalin bind to the target protein with higher affinities (–9.09 kcal/mol and –8.88 kcal/mol, respectively) than the control ligand methicillin (–6.88 kcal/mol). These two compounds have already been identified as significant constituents of *Terminalia catappa* with potential activity against methicillin-resistant *S. aureus* (MRSA). One of the key residues mediating hydrogen bonding in this study, Ser149, was also highlighted in our results. Moreover, Ser149 and Asp295—identified as important interacting partners—are part of the PBP2a allosteric domain [33]. Occupancy of this allosteric pocket by punicalagin is predicted to induce conformational changes that open PBP2a’s active site, thus facilitating β-lactam antibiotic access to the catalytic serine and effectively disabling the enzyme. Moreover, punicalagin has also been reported to suppress methicillin resistance of *S. aureus* to oxacillin [34].

Ellagic acid exhibited the strongest binding affinity to PBP3, with a binding energy of −9.01 kcal/mol. The interactions were stabilized by conventional hydrogen bonds involving Ser 392, Ser 448, and Asn 450, as shown in Figure 3a. These residues were also highlighted in a previous in silico study as critical for binding another phytochemical, emodin, which was shown to enhance synergistic activity with antibiotics and significantly reduce their required dosages [35,36].

Molecular docking results identified MurA as a potential target, because punicalin exhibited the strongest binding affinity (−8.95 kcal/mol) among the tested compounds. This interaction was observed with the MurA structure derived from *S. aureus*, representing Gram-positive bacteria. MurA catalyzes the first committed step in bacterial cell wall peptidoglycan synthesis, transferring enolpyruvate from phosphoenolpyruvate (PEP) to UDP-N-acetylglucosamine [37]. Although punicalin does not directly interact with the catalytic residue Cys 115—known to bind phosphoenolpyruvate and induce conformational changes in the adjacent loop region upon ligand binding —its hydrogen-bonding interactions with Glu 127, Thr 329, and Glu 332 may similarly trigger conformational changes [38]. These interactions could block the MurA active site by preventing the structural rearrangements required for catalysis. Inhibition of MurA would primarily affect newly synthesized peptidoglycan, leading to gradual weakening of the cell wall as the bacteria grow and divide, rather than immediate cell lysis.

FtsZ, a bacterial homolog of eukaryotic tubulin, is essential for bacterial cell division, forming a ring-like structure (Z-ring) at the prospective division site. Inhibition of FtsZ leads to filamentous growth and eventually cell death [39]. Our docking studies indicated that punicalagin binds to the FtsZ with highest affinity for both tested structures from *S. aureus* (−8.65 kcal/mol) and *E. coli* (−10.03 kcal/mol). It is noteworthy that punicalagin adopts two distinct binding site preferences in the two FtsZ crystal structures. This likely reflects differences in protein conformation and species-specific pocket geometry between 3VOB (*S. aureus* FtsZ) and 6UMK (*E. coli* FtsZ) forming hydrogen bonds with residues.

However, analysis of the binding modes reveals that punicalagin forms strong hydrogen bonds with Asn 188, Gln 192, and Lys 243 of *S. aureus* FtsZ, as well as with Gly 20, Asn 43, Gly 67, and Arg 142—residues belonging to the conserved globular core of the protein [40]. In contrast, the smaller phenolic compounds gallic acid and ellagic acid primarily interact with Val 297 and Asn 299, residues located within the hydrophobic core that plays a critical role in stabilizing dacomitinib in complex with *S. aureus* FtsZ, which has demonstrated potent inhibitory activity against MRSA strains. Moreover, Asp 199 and Thr 265 have been identified as key interaction residues, and similar binding profiles have been observed for these smaller polyphenols [41]. Additionally, withaperuvin C, a withanolide identified in *Physalis peruviana*, forms hydrogen bonds with Gly 20, Asn 43, and Asp 45 of *E. coli* FtsZ—interactions that are also shared by other natural products such as withanolide and trifolirhizin, isolated from *Glycyrrhiza pallidiflora* and *Euchresta formosana.*

Caver Web simulations demonstrated that gallic acid and ellagic acid are capable of translocating through the *E. coli* porin channel. Their favorable activation energy profiles and binding affinities within the channel indicate higher predicted permeability, suggesting an enhanced ability to penetrate the bacterial cell. In contrast, punicalin, punicalagin, and pedunculagin, when analyzed using the *E. coli* OmpF porin model, did not produce valid transport trajectories. This is likely due to their large molecular size, rigidity, and structural complexity, which exceed the dimensional and steric constraints of the porin tunnel. These computational results support the hypothesis that ellagitannins are unable to undergo passive diffusion through Gram-negative bacterial porins, implying that their antimicrobial activity may be restricted to surface-level interactions or more effective against Gram-positive organisms. These findings are consistent with in vitro data and provide a mechanistic explanation for the lower susceptibility of *E. coli* to PoPEx compounds. While smaller polyphenols likely play a dominant role in antimicrobial effects against Gram-negative bacteria, larger hydrolyzable tannins appear to be more active against Gram-positive strains. The differential antimicrobial activity of the polyphenolic compounds against Gram-positive and Gram-negative bacteria, combined with the molecular docking and transport studies, allows for the elucidation of structure–activity relationships that may guide the development of more effective antimicrobial agents.

For activity against Gram-positive bacteria, the results suggest that larger, more complex polyphenolic structures with multiple hydroxyl groups, such as punicalagin and punicalin, are advantageous. These compounds can form numerous hydrogen bonds and hydrophobic interactions with bacterial targets, enhancing their binding affinity and antimicrobial potency. The galloyl and hexahydroxydiphenoyl (HHDP) moieties present in these compounds appear to be particularly important for their antimicrobial activity, as they can engage in π-π stacking interactions with aromatic residues in protein binding sites and form hydrogen bonds with polar residues [42].

These structure–activity relationships suggest that a dual-targeting approach may be beneficial for developing broad-spectrum antimicrobial agents based on polyphenolic scaffolds. Compounds that combine the structural features of both large and small polyphenols, or formulations that include a mixture of different-sized polyphenolic compounds, may exhibit enhanced activity against both Gram-positive and Gram-negative bacteria. Additionally, strategies to enhance the permeability of the outer membrane of Gram-negative bacteria, such as the co-administration of membrane-permeabilizing agents or the development of delivery systems that can bypass the outer membrane barrier, may improve the efficacy of larger polyphenolic compounds against these pathogens.

The findings of this study highlight the potential of pomegranate peel extract and its polyphenolic constituents as natural antimicrobial agents with diverse mechanisms of action. The broad-spectrum activity of PoPEx, albeit with varying potency against different bacterial groups, suggests its potential application in various fields, including food preservation, wound management, and development of antimicrobial surfaces. In the food industry, PoPEx could serve as a natural preservative to extend the shelf life of perishable products by inhibiting bacterial growth. Its natural origin and established safety profile make it an attractive alternative to synthetic preservatives. The astringent taste of PoPEx, due to its high tannin content, may limit its application in some food products, but this could be mitigated through encapsulation technologies or by using it in foods where astringency is desirable or less noticeable. In wound management, PoPEx could be incorporated into dressings or topical formulations to prevent or treat bacterial infections, particularly those caused by Gram-positive pathogens such as *S. aureus*. The multiple mechanisms of action of PoPEx may reduce the likelihood of resistance development, a significant advantage over conventional antibiotics. Additionally, the antioxidant and anti-inflammatory properties of pomegranate polyphenols may provide additional benefits for wound healing [43].

For the development of new antimicrobial drugs, the polyphenolic compounds identified in this study, particularly punicalagin and gallic acid, could serve as lead compounds for structural optimization. Medicinal chemistry approaches could be employed to enhance their antimicrobial potency, improve their pharmacokinetic properties, and expand their spectrum of activity. For example, derivatives of gallic acid with enhanced permeability through the outer membrane of Gram-negative bacteria could be developed by modifying its carboxyl group or introducing additional functional groups that facilitate membrane penetration. Combination therapy approaches, where PoPEx or its constituents are used in conjunction with conventional antibiotics, represent another promising approach for future research. The diverse mechanisms of action of pomegranate polyphenols may complement those of existing antibiotics, potentially leading to synergistic effects and allowing for lower doses of both agents. This approach could be particularly valuable for addressing antibiotic resistance, as it may restore the efficacy of antibiotics against resistant strains and reduce the selective pressure for resistance development.

Future studies should also explore the in vivo efficacy and safety of PoPEx and its constituents in animal models of bacterial infections, as well as potential interactions with the host immune system and microbiome. The development of targeted delivery systems to enhance the bioavailability and site-specific action of these compounds would be valuable for their clinical application. Furthermore, the potential for resistance development against these natural antimicrobial agents, although theoretically lower due to their multiple mechanisms of action, should be systematically evaluated through long-term exposure studies and genetic analysis of potentially resistant strains.

## 4. Materials and Methods

### 4.1. Plant Material and Extract Preparation

Pomegranate fruits (*Punica granatum* L.) were obtained from a local market and authenticated by a botanist. The fruits were washed thoroughly with distilled water, and the peels were manually separated from the edible portions. The peels were air-dried at room temperature (25 ± 2 °C) for 14 days until a constant weight was achieved. The dried peels were ground into a fine powder using an electric grinder and stored in airtight containers at 4 °C until further use.

The extraction of bioactive compounds from pomegranate peel was performed using the maceration method. Briefly, 100 g of dried pomegranate peel powder was soaked in 1000 mL of 80% aqueous methanol (*v*/*v*) and agitated on a mechanical shaker (150 rpm) for 72 h at room temperature. The mixture was filtered through Whatman No. 1 filter paper, and the residue was re-extracted twice under the same conditions to ensure maximum extraction of bioactive compounds. The combined filtrates were concentrated under reduced pressure using a rotary evaporator (Büchi R-210, Büchi Labortechnik AG, Flawil, Switzerland) at 40 °C.

The concentrated extract was then evaporated to dryness to obtain a dry powder, which was stored at −20 °C in airtight, light-protected containers until further analysis.

### 4.2. Chemical Analysis of Polyphenolic Compounds

The methanol extract (5 mg/mL) was analyzed using liquid chromatography–mass spectrometry (LC–MS). The analysis was conducted on an Agilent Technologies HPLC 1260 Infinity (Santa Clara, CA, USA). system equipped with an autosampler, diode array detector (DAD), and a single quadrupole mass spectrometer (Single Quad MS Detector 6130, Agilent Technologies, Santa Clara, CA, USA). Chromatographic separation was achieved using a Zorbax SB-Aq C18 column (3.0 × 150 mm, 3.5 μm particle size) maintained at 25 °C. The mobile phase consisted of solvent A (0.1% formic acid in water) and solvent B (acetonitrile), applied using a binary gradient at a flow rate of 0.3 mL/min as follows: 0–30 min, 10–25% B; 30–35 min, 25–70% B; 35–40 min, return to 10% B. The injection volume was 3 μL. Detection was performed at 280 and 350 nm using DAD, and MS detection was carried out in negative ion mode over the *m*/*z* range of 50–2000.

Electrospray ionization (ESI) was performed under atmospheric pressure with the following parameters: nebulizer pressure 40 psi, drying gas temperature 350 °C, and nitrogen gas flow rate of 10 L/min. Deprotonated molecular ions and fragmentation patterns were recorded under fragmentation voltages of 100 V and 250 V in full-scan mode.

Compounds were tentatively identified by comparing their UV and MS spectra with those of commercial standards or literature data. HPLC-grade solvents were obtained from J.T. Baker (Deventer, The Netherlands). Standard compounds—ellagic acid, gallic acid, punicalagin, punicalin, and pedunculagin—were purchased from Sigma (St. Louis, MO, USA). Results were expressed as milligrams per gram of dry extract (mg/g).

### 4.3. In Vitro Antimicrobial Activity

The MICs of PoPEx and its major polyphenols were determined using the broth microdilution method on 6 clinical isolates of *S. aureus* and 5 clinical isolates of *E. coli*, representing drug-resistant Gram-positive and Gram-negative strains. Isolates were obtained from the Department of Clinical Microbiology, University Clinical Centre of the Republic of Srpska.

Bacteria were cultured on blood agar and incubated for 18 h at 37 °C. Active cultures were prepared in Mueller-Hinton broth (MHB) and adjusted to 0.5 McFarland standard (~10^6^–10^8^ CFU/mL), then diluted to 5 × 10^5^ CFU/mL for inoculation.

PoPEx and individual polyphenols were dissolved in sterile water, and twofold serial dilutions were prepared in 96-well plates to achieve final concentrations ranging from 15.62 to 500 µg/mL.

Negative controls contained non-inoculated MHB; positive controls contained bacterial suspension without extracts. Each well received 100 µL of MHB, extract solution, and 5 µL of bacterial inoculum. Plates were sealed and incubated at 37 °C for 24 h.

MIC was defined as the lowest concentration showing no visible bacterial growth (no turbidity). All tests were performed in duplicate

### 4.4. Molecular Docking Studies

The crystallographic structures of the bacterial targets—penicillin-binding proteins (PBPs; PDB IDs: 1VQQ and 3PBR), UDP-N-acetylglucosamine enolpyruvyl transferase (MurA; UniProt ID: P84058, PDB ID: 3KR6), and the cell division protein FtsZ (PDB IDs: 3VOB and 6UMK)—were retrieved from the Protein Data Bank (www.rcsb.org, accessed on 31 May 2025). Structure preparation was performed using YASARA Structure (v. 20.4.24), including the removal of water molecules, addition of hydrogen atoms, assignment of appropriate protonation states, charge optimization, and energy minimization at physiological pH (7.4) [44]. Ligands were built in 3D and energy-minimized under the same conditions using YASARA. Molecular docking was conducted using AutoDock VINA, with a blind docking protocol focused within a 5 Å radius around protein structure [45]. The best-ranked conformations based on binding energy were selected, and their interactions with target proteins were visualized and analyzed using Discovery Studio Visualizer (v.20.1.0.19295).

Molecular docking studies were performed to investigate the interactions between the major polyphenolic compounds of PoPEx (punicalagin, punicalin, ellagic acid, and gallic acid) and selected bacterial targets, including PBP2a, MurA enzyme, and FtsZ protein. The three-dimensional structures of the polyphenolic compounds were obtained from the PubChem database (https://pubchem.ncbi.nlm.nih.gov/, accessed on 19 May 2025) and optimized using the MMFF94 force field in Avogadro software.

### 4.5. Transport Studies Through Porin Channels

To investigate the translocation of polyphenolic compounds from pomegranate peel extract (PoPEx) through Gram-negative outer membrane channels, transport simulations were performed using Caver Web v. 2.0 The crystal structure of *E. coli* porin OmpF (PDB ID: 2OMF) was prepared by removing non-essential molecules, adding hydrogens, and assigning protonation states at pH 7.4. Representative PoPEx compounds (e.g., punicalagin, ellagic acid) were energy-minimized, converted to 3D structures, and protonated appropriately before being saved in PDBQT format.

Tunnels within the OmpF structure were detected using Caver Web v. 2.0, and the primary transmembrane pore was selected and discretized into slices to serve as a ligand pathway. Caver simulations with polyphenolic compounds were conducted in the extracellular-to-intracellular direction, with the ligands incrementally docked along the tunnel using a restrained AutoDock Vina-based approach. The resulting energy profiles represent the predicted free energy of ligand movement through the pore. Transport trajectories and energy barriers were visualized and analyzed using using the integrated service of Caver Web 2.0, enabling comparison of permeability potential among tested compounds.

## 5. Conclusions

In conclusion, this study demonstrated the antimicrobial potential of PoPEx and its major polyphenolic constituents in in vitro assays, with particularly strong effects observed against *S. aureus* clinical isolates. Chemical analysis confirmed the presence of punicalagin, punicalin, pedunculagin, ellagic acid, and gallic acid, and their differential activity was consistent with structural differences in bacterial envelopes and compound size. Larger ellagitannins showed greater activity against Gram-positive isolates, whereas smaller phenolics such as gallic acid exhibited enhanced effects against *E. coli*, in line with membrane permeability constraints. Molecular docking and transport simulations further supported these in vitro findings by indicating distinct interactions with bacterial targets. While these results highlight the promise of PoPEx as a natural antimicrobial source, they remain preliminary, and further studies are needed to assess extract standardization, bioavailability, and in vivo efficacy.

## Figures and Tables

**Table 1:** Quantification of major polyphenolic compounds in PoPEx.

No.	RT (280 nm)	MW	[M − H] − (*m*/*z*) (100 V)	MS Data (*m*/*z*) (250V)	Compound Name	Concentration (mg/g Dry Extract)
1	3235	482	481	275, 301	HHDP-hexoside	4.58 ± 0.00
2	3736	782	781	601, 721	Punicalin isomer α (4,6-gallagyl-glucose)	10.44 ± 0.00
3	3933	782	781	601, 721	Punicalin isomer β	12.14 ± 0.00
4	5045	784	783	481, 301	Penduculagin I isomer (bis-HHDP-hexoside)	12.5 ± 0.00
5	8329	1084	1083	781, 601	Punicalagin isomer α (HHDP-gallagyl-glicoside)	311.28 ± 0.23
6	11,064	1084	1083	781, 601	Punicalagin isomer β (HHDP-gallagyl-glucoside)	312.63 ± 0.28
7	13,795	634	633	463, 301	Galloyl–HHDP hexoside	5.71 ± 0.00
8	15,373	786	785	755, 301	Tellimagrandin I	2.74 ± 0.00
9	17,343	464	463	301, 463	Ellagic acid-hexoside	6.01 ± 0.01
10	25,177	302	301	301	Ellagic acid	26.54 ± 0.03

**Table 2:** Antibiotic susceptibility profiles of clinical isolates of *S. aureus* and *E. coli*.

Isolate Code	Species	Penicillin	Ampicillin	Amoxicillin/Clavulanic Acid	Cefaclor	Ceftriaxone	Erythromycin	Gentamicin	Ciprofloxacin	Vancomycin	Cefuroxime	Cefotaxime	Ceftazidime	Cefepime	Amikacin	Trimethoprim/Sulfamethoxazole	Piperacillin	Piperacillin/Tazobactam	Imipenem	Meropenem
1	***S. aureus***	S	S	S	S	S	S	S	S	S	-	-	-	-	-	-	-	-	-	-
2		R	R	S	S	S	S	S	R	S	-	-	-	-	-	-	-	-	-	-
3		S	S	S	S	S	S	S	S	S	-	-	-	-	-	-	-	-	-	-
4		S	S	S	S	S	R	S	S	S	-	-	-	-	-	-	-	-	-	-
5		S	S	S	S	S	S	R	S	S	-	-	-	-	-	-	-	-	-	-
6		S	S	S	S	S	S	S	S	S	-	-	-	-	-	-	-	-	-	-
1	***E. coli***	-	R	R	-	S	-	S	S	-	S	S	S	S	S	S	R	S	S	S
2		-	R	S	-	S	-	S	S	-	S	S	S	S	S	S	R	S	S	S
3		-	R	S	-	S	-	S	S	-	S	S	S	S	S	S	R	S	S	S
4		-	S	S	-	S	-	S	R	-	S	S	S	S	S	R	R	S	S	S
5		-	R	R	-	S	-	R	S	-	S	S	S	S	S	R	R	S	S	S

S = Sensitive (susceptible); R = Resistant; Empty cell (-) = Not tested.

**Table 3:** Minimum inhibitory concentration (MIC) values of PoPEx and individual polyphenols against clinical isolates of *S. aureus* (µg/mL).

Isolate	PoPEx	Punicalin	Punicalagin	Pedunculagin	Gallic Acid	Ellagic Acid
1	31.25 ± 0.00	62.50 ± 0.00	15.62 ± 0.00	500.00 ± 0.00	125.00 ± 0.00	250.00 ± 0.00
2	31.25 ± 0.00	62.50 ± 0.00	20.83 ± 7.37	>500.00	125.00 ± 0.00	250.00 ± 0.00
3	20.83 ± 7.37	62.50 ± 0.00	15.62 ± 0.00	500.00 ± 0.00	125.00 ± 0.00	250.00 ± 0.00
4	31.25 ± 0.00	52.08 ± 17.73	15.62 ± 0.00	500.00 ± 0.00	125.00 ± 0.00	250.00 ± 0.00
5	31.25 ± 0.00	62.50 ± 0.00	31.25 ± 0.00	416.66 ± 117.85	125.00 ± 0.00	250.00 ± 0.00
6	20.83 ± 7.37	62.50 ± 0.00	15.62 ± 0.00	>500.00	166.67 ± 58.93	250.00 ± 0.00

**Table 4:** MIC values of PoPEx and individual polyphenols against clinical isolates of *E. coli* (µg/mL).

Isolate	PoPEx	Punicalin	Punicalagin	Pedunculagin	Gallic Acid	Ellagic Acid
1	250.00 ± 0.00	500.00 ± 0.00	250.00 ± 0.00	>500	125.00 ± 0.00	208.33 ± 58.93
2	250.00 ± 0.00	500.00 ± 0.00	333.33 ± 117.85	>500	125.00 ± 0.00	250.00 ± 0.00
3	250.00 ± 0.00	500.00 ± 0.00	250.00 ± 0.00	500.00 ± 0.00	125.00 ± 0.00	250.00 ± 0.00
4	208.33 ± 58.93	500.00 ± 0.00	250.00 ± 0.00	>500	125.00 ± 0.00	125.00 ± 0.00
5	250.00 ± 0.00	416.67 ± 117.85	250.00 ± 0.00	>500	125.00 ± 0.00	250.00 ± 0.00

**Table 5:** Molecular docking results: binding energies and interactions involved in the stabilization of the most stable complexes.

Ligand	Target Protein	GP Binding Energy (kcal/mol)	GP Interactions	GN Binding Energy (kcal/mol)	GN Interactions
Punicalin	PBPs *	−8.878	Asn 111, Asp 128, Gly 135, His 311	−6.253	Ser 429, Asn 450, Glu 623, Val 632, Val 658, Pro 659, Pro 660
Punicalagin		−9.096	Ser 149, Thr 216, Pro 258, Val 277, Asp 295, Tyr 373	−3.18	Lys 427, Arg 428, Ser 429, Asn 450, Asp 519, Glu 623, Pro 659, Pro 660
Pedunculagin		−8.659	Tyr 255, Asn 260, Glu 263, Phe 371, Asn 377, Asn 381	−6.658	Thr 426, Asn 432, Asp 519, Thr 621, Glu 623, Pro 659
Gallic a.		−5.593	Thr 444, Asn 464, Tyr 519, Glu 602	−6.074	Pro 660
Ellagic a.		−7.654	Lys 215, Val 217, Lys 218, Asp 221, Pro 370, Gly 374, Glu 379	−9.01	Ser 392, Ser 448, Asn 450, Thr 603, Gly 620, Pro 660
Punicalin	MurA **	−8.951	Pro 125, Glu 127, Phe 164, Thr 329, Glu 332	−8.841	Lys 46, Leu 47, Asp 51, Asp 396, Ile 402, Arg 415
Punicalagin		−7.765	Asp 163, Phe 164, Glu 332, Arg 353	−8.738	Lys 46, Leu 47, Asp 51, Asp 396, Arg 401, Arg 415, Lys 417
Pedunculagin		−8.434	Asp 2, Glu 52, Leu 395, Asp 399, Arg 400, Arg 418, Asn 420	−8.484	Lys 160, Val 161, Thr 326, Glu 329
Gallic a.		−6.325	Asp 308, Leu 373, Arg 400	−5.258	Pro 9, Ser 245, Arg 246, Ile 382
Ellagic a.		−8.788	Lys 22, Cys 119, Arg 124, Asp 308, Arg 334, Asp 372, Leu 373, Arg 400	−7.034	Leu 47, Tyr 399, Ile 402, Glu 403, Arg 401, Arg 415
Punicalin	FtsZ ***	−7.803	Glu 185, Asn 188, Gln 192, Lys 243, Ser 246, Pro 248	−9.154	Asn 24, Asp 45, Thr 65, Arg 142
Punicalagin		−8.648	Glu 185, Gly 229, Val 230, Lys 243, Ser 246, Pro 248	−10.031	Gly 20, Asn 43, Asp 45, Ala 48, Thr 65, Gly 67, Ala 72, Lys 141, Arg 142
Pedunculagin		−8.551	Asn 44, Asp 46, Gln 48, Ala 49	−8.658	Gly 20, Asn 24, Thr 44, Asp 45, Gly 71, Thr 108
Gallic a.		−5.426	Asp 199, Leu 200, Val 203, Val 297, Asn 299	−5.347	Gln 194, Ser 227
Ellagic a.		−8.439	Asp 199, Leu 200, Val 203, Thr 265, Val 297, Asn 299, Val 307	−7.607	Asp 187, Lys 190, Gln 194, Ser 227, Thr 309

Control ligands and their binding affinities: Methicillin * (−6.88 kcal/mol), Fosfomycin ** (−4.64 kcal/mol), and PC190723 *** (−6.805 kcal/mol).

## Data Availability

All data are available upon request.

## References

[1] WHO 2023 Antibacterial Agents in Clinical and Preclinical Development: An Overview and Analysis. https://www.who.int/publications/i/item/9789240094000.

[2] Ge S., Duo L., Wang J., GegenZhula, Yang J., Li Z., Tu Y. (2021). A unique understanding of traditional medicine of pomegranate, *Punica granatum* L. and its current research status. J. Ethnopharmacol..

[3] Grabez M., Skrbic R., Stojiljkovic M.P., Vucic V., Rudic Grujic V., Jakovljevic V., Djuric D.M., Surucic R., Savikin K., Bigovic D. (2022). A prospective, randomized, double-blind, placebo-controlled trial of polyphenols on the outcomes of inflammatory factors and oxidative stress in patients with type 2 diabetes mellitus. Rev. Cardiovasc. Med..

[4] Suručić R., Tubic B., Stojiljkovic M.P., Djuric D.M., Travar M., Grabez M., Savikin K., Skrbic R. (2021). Computational study of pomegranate peel extract polyphenols as potential inhibitors of SARS-CoV-2 virus internalization. Mol. Cell. Biochem..

[5] Suručić R., Travar M., Petković M., Tubić B., Stojiljković M.P., Grabež M., Šavikin K., Zdunić G., Škrbić R. (2021). Pomegranate peel extract polyphenols attenuate the SARS-CoV-2 S-glycoprotein binding ability to ACE2 Receptor: In silico and in vitro studies. Bioorg. Chem..

[6] Marra F., Petrovicova B., Canino F., Maffia A., Mallamaci C., Muscolo A. (2022). Pomegranate Wastes Are Rich in Bioactive Compounds with Potential Benefit on Human Health. Molecules.

[7] Singh J., Kaur H.P., Verma A., Chahal A.S., Jajoria K., Rasane P., Kaur S., Kaur J., Gunjal M., Ercisli S. (2023). Pomegranate Peel Phytochemistry, Pharmacological Properties, Methods of Extraction, and Its Application: A Comprehensive Review. ACS Omega.

[8] Saparbekova A.A., Kantureyeva G.O., Kudasova D.E., Konarbayeva Z.K., Latif A.S. (2023). Potential of phenolic compounds from pomegranate (*Punica granatum* L.) by-product with significant antioxidant and therapeutic effects: A narrative review. Saudi J. Biol. Sci..

[9] Alvarez-Martinez F.J., Barrajon-Catalan E., Encinar J.A., Rodriguez-Diaz J.C., Micol V. (2020). Antimicrobial Capacity of Plant Polyphenols against Gram-positive Bacteria: A Comprehensive Review. Curr. Med. Chem..

[10] Zhao Y., Wei J., Li C., Ahmed A.F., Liu Z., Ma C. (2022). A comprehensive review on mechanism of natural products against *Staphylococcus aureus*. J. Future Foods.

[11] Daglia M. (2012). Polyphenols as antimicrobial agents. Curr. Opin. Biotechnol..

[12] Radović J., Suručić R., Niketić M., Kundaković-Vasović T. (2022). Alchemilla viridiflora Rothm.: The potent natural inhibitor of angiotensin I-converting enzyme. Mol. Cell. Biochem..

[13] Suručić R., Radović Selgrad J., Kundaković-Vasović T., Lazović B., Travar M., Suručić L., Škrbić R. (2022). In Silico and In Vitro Studies of *Alchemilla viridiflora* Rothm—Polyphenols’ Potential for Inhibition of SARS-CoV-2 Internalization. Molecules.

[14] Bertonha A.F., Silva C.C.L., Shirakawa K.T., Trindade D.M., Dessen A. (2023). Penicillin-binding protein (PBP) inhibitor development: A 10-year chemical perspective. Exp. Biol. Med..

[15] Brdová D., Ruml T., Viktorová J. (2024). Mechanism of staphylococcal resistance to clinically relevant antibiotics. Drug Resist. Updates.

[16] Sethuvel D.P.M., Bakthavatchalam Y.D., Karthik M., Irulappan M., Shrivastava R., Periasamy H., Veeraraghavan B. (2023). β-Lactam Resistance in ESKAPE Pathogens Mediated Through Modifications in Penicillin-Binding Proteins: An Overview. Infect. Dis. Ther..

[17] Gautam A., Rishi P., Tewari R. (2011). UDP-N-acetylglucosamine enolpyruvyl transferase as a potential target for antibacterial chemotherapy: Recent developments. Appl. Microbiol. Biotechnol..

[18] Casiraghi A., Suigo L., Valoti E., Straniero V. (2020). Targeting Bacterial Cell Division: A Binding Site-Centered Approach to the Most Promising Inhibitors of the Essential Protein FtsZ. Antibiotics.

[19] Silhavy T.J., Kahne D., Walker S. (2010). The bacterial cell envelope. Cold Spring Harb. Perspect. Biol..

[20] Galdiero S., Falanga A., Cantisani M., Tarallo R., Della Pepa M.E., D’Oriano V., Galdiero M. (2012). Microbe-host interactions: Structure and role of Gram-negative bacterial porins. Curr. Protein Pept. Sci..

[21] Jenkins S.G., Schuetz A.N. (2012). Current concepts in laboratory testing to guide antimicrobial therapy. Mayo Clin. Proc..

[22] Persuric Z., Saftic Martinovic L., Malenica M., Gobin I., Pedisic S., Dragovic-Uzelac V., Kraljevic Pavelic S. (2020). Assessment of the Biological Activity and Phenolic Composition of Ethanol Extracts of Pomegranate (*Punica granatum* L.) Peels. Molecules.

[23] Colic M., Mihajlovic D., Bekic M., Markovic M., Dragisic B., Tomic S., Miljus N., Savikin K., Skrbic R. (2022). Immunomodulatory Activity of Punicalagin, Punicalin, and Ellagic Acid Differs from the Effect of Pomegranate Peel Extract. Molecules.

[24] Man G., Xu L., Wang Y., Liao X., Xu Z. (2021). Profiling Phenolic Composition in Pomegranate Peel from Nine Selected Cultivars Using UHPLC-QTOF-MS and UPLC-QQQ-MS. Front. Nutr..

[25] Liu Y., Kong K.W., Wu D.T., Liu H.Y., Li H.B., Zhang J.R., Gan R.Y. (2022). Pomegranate peel-derived punicalagin: Ultrasonic-assisted extraction, purification, and its alpha-glucosidase inhibitory mechanism. Food Chem..

[26] Qin G., Xu C., Ming R., Tang H., Guyot R., Kramer E.M., Hu Y., Yi X., Qi Y., Xu X. (2017). The pomegranate (*Punica granatum* L.) genome and the genomics of punicalagin biosynthesis. Plant J..

[27] Tang Z., Shi L., Liang S., Yin J., Dong W., Zou C., Xu Y. (2024). Recent Advances of Tannase: Production, Characterization, Purification, and Application in the Tea Industry. Foods.

[28] Henis Y., Tagari H., Volcani R. (1964). Effect of Water Extracts of Carob Pods, Tannic Acid, and Their Derivatives on the Morphology and Growth of Microorganisms. Appl. Microbiol..

[29] Engels C., Schieber A., Ganzle M.G. (2011). Inhibitory spectra and modes of antimicrobial action of gallotannins from mango kernels (*Mangifera indica* L.). Appl. Environ. Microbiol..

[30] Winterhalter M., Ceccarelli M. (2015). Physical methods to quantify small antibiotic molecules uptake into Gram-negative bacteria. Eur. J. Pharm. Biopharm..

[31] Boi S., Puxeddu S., Delogu I., Farci D., Piano D., Manzin A., Ceccarelli M., Angius F., Scorciapino M.A., Milenkovic S. (2025). Seeking Correlation Among Porin Permeabilities and Minimum Inhibitory Concentrations Through Machine Learning: A Promising Route to the Essential Molecular Descriptors. Molecules.

[32] Fishovitz J., Hermoso J.A., Chang M., Mobashery S. (2014). Penicillin-binding protein 2a of methicillin-resistant *Staphylococcus aureus*. IUBMB Life.

[33] Guan S., Zhong L., Yu H., Wang L., Jin Y., Liu J., Xiang H., Yu H., Wang L., Wang D. (2022). Molecular docking and proteomics reveals the synergistic antibacterial mechanism of theaflavin with beta-lactam antibiotics against MRSA. Front. Microbiol..

[34] Mun S.H., Kang O.H., Kong R., Zhou T., Kim S.A., Shin D.W., Kwon D.Y. (2018). Punicalagin suppresses methicillin resistance of *Staphylococcus aureus* to oxacillin. J. Pharmacol. Sci..

[35] Rolta R., Salaria D., Kumar V., Patel C.N., Sourirajan A., Baumler D.J., Dev K. (2022). Molecular docking studies of phytocompounds of Rheum emodi Wall with proteins responsible for antibiotic resistance in bacterial and fungal pathogens: In silico approach to enhance the bio-availability of antibiotics. J. Biomol. Struct. Dyn..

[36] Rolta R., Kumar V., Sourirajan A., Upadhyay N.K., Dev K. (2020). Bioassay guided fractionation of rhizome extract of Rheum emodi wall as bio-availability enhancer of antibiotics against bacterial and fungal pathogens. J. Ethnopharmacol..

[37] Kock H., Gerth U., Hecker M. (2004). MurAA, catalysing the first committed step in peptidoglycan biosynthesis, is a target of Clp-dependent proteolysis in *Bacillus subtilis*. Mol. Microbiol..

[38] Zhu J.Y., Yang Y., Han H., Betzi S., Olesen S.H., Marsilio F., Schonbrunn E. (2012). Functional consequence of covalent reaction of phosphoenolpyruvate with UDP-N-acetylglucosamine 1-carboxyvinyltransferase (MurA). J. Biol. Chem..

[39] Szwedziak P., Wang Q., Bharat T.A., Tsim M., Lowe J. (2014). Architecture of the ring formed by the tubulin homologue FtsZ in bacterial cell division. eLife.

[40] Pradhan P., Margolin W., Beuria T.K. (2021). Targeting the Achilles Heel of FtsZ: The Interdomain Cleft. Front. Microbiol..

[41] Du R.L., Sun N., Fung Y.H., Zheng Y.Y., Chen Y.W., Chan P.H., Wong W.L., Wong K.Y. (2022). Discovery of FtsZ inhibitors by virtual screening as antibacterial agents and study of the inhibition mechanism. RSC Med. Chem..

[42] Pinheiro A., Mendes A.R.S., Neves M., Prado C.M., Bittencourt-Mernak M.I., Santana F.P.R., Lago J.H.G., de Sa J.C., da Rocha C.Q., de Sousa E.M. (2019). Corrigendum: Galloyl-Hexahydroxydiphenoyl (HHDP)-Glucose Isolated from *Punica granatum* L. Leaves Protects Against Lipopolysaccharide (LPS)-Induced Acute Lung Injury in BALB/c Mice. Front. Immunol..

[43] Yousefian F., Hesari R., Jensen T., Obagi S., Rgeai A., Damiani G., Bunick C.G., Grada A. (2023). Antimicrobial Wound Dressings: A Concise Review for Clinicians. Antibiotics.

[44] Land H., Humble M.S. (2018). YASARA: A Tool to Obtain Structural Guidance in Biocatalytic Investigations. Methods Mol. Biol..

[45] Trott O., Olson A.J. (2010). AutoDock Vina: Improving the speed and accuracy of docking with a new scoring function, efficient optimization, and multithreading. J. Comput. Chem..

